# Phonetic Development of an L2 Vowel System and Tandem Drift in the L1: A Residence Abroad and L1 Re-Immersion Study

**DOI:** 10.1177/00238309221133100

**Published:** 2022-11-22

**Authors:** James Turner

**Affiliations:** University of Southampton, UK

**Keywords:** Phonetic drift, L2 speech learning, L2 development, L1 re-immersion, speech plasticity, attrition

## Abstract

This study analyzes the production of native (L1) and foreign (L2) vowels by 42 L1 English learners of French (ELoF) at the start and end of a 6-month residence abroad (RA) in a French-speaking country. Data are also reported from a delayed post-test, which takes place 10 months after a subsection of participants (*n* = 27) return to the L1 English environment. Results reveal systemic *phonetic drift* in ELoF’s L1 English vowels over the RA, and this accompanies the phonetic development occurring in the participants’ L2 French vowel system, a phenomenon we label “tandem drift.” This L1–L2 link is also supported by interspeaker variation: the individuals whose L2 French vowels shift the most are also the participants who exhibit the most substantial L1 phonetic drift in the same direction. Results for the L1 re-immersion time point suggest a partial—but not complete—reversal of phonetic drift, whereas no reversal of the L2 gains made over the RA is apparent. Nevertheless, at the individual level, the learners whose L2 gains reverse the most upon L1 re-immersion are also most likely to exhibit reverse phonetic drift in their L1. Overall, these findings indicate a relationship between L2 speech learning and L1 phonetic drift, which we argue is driven by the global phonetic properties of both L2 and L1 becoming linked at a representational level. Although these representations appear malleable, it is clear that recent changes are not guaranteed to reverse despite substantial re-exposure to L1 input. Implications for the distinction between drift and attrition are discussed.

## 1 Introduction

Studies have shown that the properties of native (L1) speech can be influenced by contact with a foreign language (L2) not only after long periods of immersion in an L2 environment, a process known as *phonetic attrition* (Bergmann et al., 2016; De Leeuw et al., 2012; Mayr et al., 2012; Stoehr et al., 2017), but also within only weeks of intensive L2 input, a phenomenon sometimes referred to as *phonetic drift* (e.g., Chang, 2012, 2013).

Although these two terms are not straightforwardly distinguishable, phonetic attrition is argued to be characterized by long-term effects of L2 exposure, which “last in the absence of a proximal L2 stimulus” (Chang, 2019b, p. 202). Furthermore, studies analyzing this process are often limited to cross-sectional analyses with potential attriters compared with monolinguals (although, see De Leeuw, 2019, and Kornder & Mennen, 2021, for notable exceptions involving real-time analyses). Phonetic drift, however, is deemed a response to *recent* L2 input, can often be tracked longitudinally, and is argued to be more reversible upon L1 re-immersion (see Chang, 2019b, for more on this discussion).

The present research concerns itself with the latter of these processes, “phonetic drift.” To date, it is unclear whether the rapid change observed in L1 productions over time is linked to the development occurring in language learners’ foreign sounds, that is, recent L2 acquisition, or whether exposure to a foreign language (irrespective of whether that exposure translates to L2 production gains) leads to phonetic drift toward the average acoustic properties of the foreign language input (henceforth “L2 norms”). Furthermore, although phonetic drift is deemed reversible, the persistence of these phonetic changes upon L1 re-immersion has yet to be thoroughly tested (Chang, 2019b, p. 202).

The present research addresses these concerns with a real-time analysis of both L1 and L2 acoustic vowel data over a residence abroad (RA) and at a delayed post-test conducted 10 months after participants return home to the L1 environment. Our results have broader implications for theories of how native and foreign sounds are stored by language learners, as well as furthering our understanding of phonetic plasticity in the face of varying ambient linguistic inputs.

## 2 Background

### 2.1 Phonetic drift and the SLM

One of the only L2 speech models to date which focuses on the malleability of L1 phonetic categories in response to L2 learning is the *speech learning model* (*SLM*) (Flege, 1995, 2007) and its revised version, the *SLM-r* (Flege & Bohn, 2021). Although much of the data informing the *SLM* are collected from second language learners who have lived in the L2 environment for many years, the *SLM* has been used liberally as a conceptual framework for studies of recent L1 phonetic changes such as those observed in instances of phonetic drift (e.g., Chang, 2012). According to the model, changes to the L1 phonetic inventory can be observed in one of two scenarios.

The first is *phonetic category assimilation* (Flege, 2007, p. 367; Flege & Bohn, 2021, p. 41). In such instances, an L2 sound is perceived as phonetically similar to an L1 sound, and this impedes the development of a new phonetic category. This, in turn, may lead to both L2 and L1 phones approximating each other in production under a “composite” category, which is predicted to ultimately resemble the “combined distributions of the perceptually linked L1 and L2 sounds” (Flege & Bohn, 2021, p. 41).

The second scenario is when *phonetic category dissimilation* occurs, marking the development of a new category (Flege, 2007, p. 370; Flege & Bohn, 2021, p. 42). In such instances, a speaker’s L1 and L2 sounds may disperse to avoid overlap within a singular phonetic space (Flege & Bohn, 2021, p. 42) and because both L1 sounds and L2 sounds have the potential to deflect (Flege, 2007, p. 371), a native sound may drift away from a similar L2 sound which is undergoing development.

It would appear, then, that if phonetic drift is observed in instances where no L2 development occurs, the *SLM-r* suggests assimilatory drift between L1 and L2 productions will be the most common outcome. However, if L2 phonetic gains are observed, phonetic drift is more likely to be dissimilatory in nature, away from the learners’ developing L2 categories. Nevertheless, debates remain as to whether assimilation between learners’ L1 and L2 categories is uniquely reserved for cases in which the formation of new phonetic categories has been severely inhibited, or whether it can still occur in instances of L2 phonetic gains. Similarly, it is unclear whether dissimilatory drift between L1 and L2 categories can only occur in instances of L2 phonetic development, or whether the L1 sound can shift away from a similar L2 sound whose development appears blocked. In the following section, we revisit the notions of assimilation and dissimilation with reference to previous longitudinal drift studies in which both L1 and L2 speech data are reported.

### 2.2 Phonetic category assimilation and dissimilation in phonetic drift research

Phonetic category assimilation (Flege, 1995, 2007; Flege & Bohn, 2021) has been observed in several previous studies of longitudinal phonetic drift. For example, Chang (2012) analyzes the speech of L1 American English speakers who are enrolled in an L2 Korean course and stay at a South Korean university. The participants are recorded producing items in English and Korean over the first 5 weeks of the Korean course. In the females’ native English stops, fundamental frequency (F0) at the onset of the following vowel increases over the sessions, approaching F0 following their Korean fortis and aspirated stops. Because F0 in their Korean productions does not increase in line with their English F0, however, the difference in F0 between their production of the two languages decreases, consistent with phonetic category assimilation. No significant change was observed in the onset of F0 in either language among the male participants, however, demonstrating that the lack of L2 development does not necessitate phonetic category assimilation.

Another example of phonetic category assimilation can be found in Sancier and Fowler (1997). In this study, an adult L1 Brazilian Portuguese/L2 English speaker was recorded after residing 4.5 months in the United States, again at the end of a 2.5-month stay in Brazil, and finally, 4 months after her return to the United States. As the participant moved from Brazil to the United States, the voice onset time (VOT) of her L1 Portuguese /t/ elongated to become more English-like, whereas no lengthening in VOT occurred for her L2 English /t/, resulting in the acoustic difference between the learners’ L1 and L2 productions diminishing.

Although the *SLM-r* theory of phonetic category assimilation appears to be supported by these phonetic drift studies, the model also suggests that phonetic category dissimilation will only occur if new L2 phonetic categories are under formation. However, few if any *longitudinal* short-term phonetic studies to date have documented cases of dissimilatory drift between L1 and L2 categories. Instead, when real-time changes occur in L2 production, potentially signifying the development of a new category, often this leads to accompanying drift in the L1 counterpart rather than a deflection away from the L2 sound.

For example, although phonetic category assimilation appears to occur for F0 in Chang (2012), this is not the case for VOT. Instead, the change in VOT of stops in both languages occurs in tandem: as the VOT of learners’ L2 Korean aspirated stops lengthens, the VOT of participants’ L1 English voiceless stops also increases. Similarly in Sancier and Fowler (1997), phonetic category assimilation does not appear to occur in the speaker’s /p/ production: the VOT of her L1 Portuguese /p/ increases to become more English-like, but this is accompanied by the lengthening of VOT for the speaker’s L2 English /p/. Instead, we introduce the term “tandem drift” for such instances.

### 2.3 Tandem phonetic drift

We reserve the term tandem drift for cases of phonetic drift in which the phonetic change observed in the L1 accompanies the development of a similar sound or set of sounds in language learners’ L2 production. In instances of tandem drift, the acoustic properties of both L1 and L2 shift significantly in the same direction over a given period and, more often than not, the phonetic change in both languages will converge on the norms of the L2 input. However, because L2 development does not always follow a linear path (see, for example, Abrahamsson, 2003), language learners’ L2 productions may progress toward, then regress away from an L2 target, as well as undershoot or overshoot L2 norms. As such, if phonetic drift occurs in tandem with L2 acquisition, these L1 phonetic changes may not always converge on the values of the L2 input, but instead follow the fluctuating trajectory of the learners’ L2 system.

The reason that the expected direction of phonetic change in both L1 and L2 is, nonetheless, more likely to be toward the norms of the L2 input is due to cross-linguistic influence in the initial stages of L2 learning. For example, a native Spanish speaker beginning to learn English may produce their L2 English voiceless stops with shorter VOTs than the English norms because Spanish features short-lag stops while English features long-lag stops. As such, both the learner’s L1 and L2 sounds are shorter than the L2 norm, which would predict that phonetic change in both systems will manifest itself as VOT lengthening toward the same L2 target. In such instances, there are inherent difficulties in determining whether L1 phonetic drift arises from contact with a new ambient linguistic input, or a consequence of actual development in L2 pronunciation. This is noted by Chang (2019b, p. 202): “it is often [. . .] impossible to tease apart the effect of L2 exposure from the role of L2 acquisition.”

The results of Kartushina and Martin (2019) provide another fitting example. Here, the authors analyze the vowel productions of Spanish-Basque bilinguals partaking in a study abroad scheme in the Netherlands to improve their (L3) English. Patterns at the group level reveal a significant lowering of the learners’ Basque and Spanish vowel spaces toward the average acoustic values of the English input. However, this lowering also accompanies a systemic change in the learners’ L3 English vowels, which shift to become more target-like. These findings appear to exemplify tandem drift, but it is unclear whether the L1 change is driven by exposure to the English input alone or the development occurring in the learners’ pronunciation of English vowels.

L2 pronunciation training studies have stressed the importance of considering L2 acquisition as a determinant factor, rather than exposure per se, by demonstrating that phonetic drift can accompany L2 gains even in an L1 environment. For example, Kartushina et al. (2016) report that L1 French speakers who receive training on Russian /ɨ/ not only alter their L2 productions to approximate the L2 Russian target but also exhibit phonetic drift in their L1 productions of French /ø/ and to a lesser extent, /y/. Furthermore, at an individual level, participants whose L2 Danish /ɔ/ changes most in terms of F1 and F2 after training are also most likely to alter their L1 French /o/ in a similar direction. The fact that phonetic drift can occur in L1-immersed classroom learners is also supported by recent cross-sectional studies in which the VOT of classroom language learners is found to be significantly different from monolingual controls (e.g., Dmitrieva et al., 2020; Osborne & Simonet, 2021).

Although several studies indicate that acquisition of a similar L2 vowel may encourage phonetic drift of an L1 vowel, other research has found that phonetic drift is not determined on a segment-by-segment basis but, rather, at a systemic level.

### 2.4 Systemic phonetic drift in the L1 vowel space

One pioneering study to investigate phonetic interactions between vocalic systems is that of Guion (2003).^1^ That study analyzes the vowels of L1 Quichua-L2 Spanish speakers in relation to monolingual Spanish speakers in the knowledge that Quichua high vowels are generally lower than those in Spanish, whereas the Quichua low vowel is generally higher than the Spanish equivalent. Findings reveal that the L1 Quichua vowels of early Spanish L2 learners have lower F1 values than those of late Spanish L2 learners, indicating that as L2 Spanish experience increases, speakers adapt their Quichua vowel space upward. Because not all Spanish vowels are higher than in Quichua, however, this upward shift cannot be explained by segment-by-segment mergers between the two languages. Instead, the author proposes that incorporating Spanish /e/, /o/ and /a/ within a bilingual’s existing sound system puts pressure on the Quichua vowels to become more raised at a systemic level, increasing the cross-linguistic distinction.

However, this dispersion effect is not consistent with the findings in Chang (2012). In that study, the native English vowels of the L1 English-L2 Korean female speakers drift toward the L2 Korean norms, resulting in decreased cross-linguistic dispersion, rather than the predicted increase. Instead, Chang (2012) suggests that because the average F1 of the Korean vowel inventory is lower than that of the learners’ native English system, drift toward the global formant values of the ambient L2 input results in L1 vowel raising. Other recent phonetic drift studies (e.g., Chang, 2019a; Kartushina & Martin, 2019; Lang & Davidson, 2019) are also consistent with Chang’s proposal that global formant values in the L2 input determine the direction of the L1 vocalic drift. Nevertheless, given the limited number of longitudinal vocalic studies, the direction of phonetic drift clearly warrants further exploration.

### 2.5 Effects of L1 re-immersion on L1 and L2 phonetic categories

A further open question regarding phonetic drift is the persistence of L1 changes upon returning home and whether this is affected by the reversal of L2 gains. Very few phonetic studies to date have tracked language learners as they move from an L2 environment back to their native language environment, and even fewer contain baseline data of L1 pronunciation before participants moved to the L2 environment in the first place. As such, Chang (2019b, p. 202) notes,

it is often unclear whether observed L1 changes are short-term, long-term, or medium-term (i.e., reversible, but not quickly or easily).

A handful of studies have, nevertheless, focused on the reversibility of drift. One example is the early pioneering work of Sancier and Fowler (1997), summarized earlier. As an L1 Portuguese speaker moves from the ambient L2 English environment to her native language environment, both the speaker’s L1 Portuguese /p/ and /t/ VOTs shorten, a finding consistent with the notion that productions “reset” to the L1 norms after L1 re-immersion. Similarly, the participant’s L2 English VOT values for /p/ and /t/ shorten, suggesting that L2 acquisition is also unstable. Interestingly, the distance between L1 and L2 categories diminishes for both /p/ and /t/, indicating phonetic category assimilation may occur over time.

Results from Tobin et al. (2017) also support the notion that the difference between the learners’ L1 and L2 productions lessens. Here, the authors analyze the VOT of voiceless stops among 10 native Spanish speakers at two time points: 4 months after living in the United States and 2–4 weeks after returning to Spain. Although the L2 English VOT values become shorter after re-immersion in the L1 Spanish-speaking environment, echoing results in Sancier and Fowler (1997), no change to the learners’ L1 Spanish VOTs is observed from renewed contact with L1. As such, the L1–L2 VOT difference decreases, a finding consistent with phonetic category assimilation.

Other studies have shown that L1 re-immersion can affect both learners’ native and foreign sounds to a similar extent. For example, in Kartushina and Martin (2019), also summarized earlier, Spanish-Basque bilinguals were recorded both 1 day after their return to Spain from their English course in the Netherlands and 4 months after their return. Findings suggest that the systemic lowering exhibited in the participants’ native vowel space over the participants’ 2 weeks abroad, as well as the changes exhibited in the L3 English vowel space are entirely reversed upon 4 months of re-immersion, with no significant F1 differences from the pre-test. Qualitative assessment of these patterns suggests that the acoustic distance between learners’ native and foreign vowel systems does not change: participants’ native and foreign sounds undergo reverse drift to a similar extent.

Based on this brief overview of L1 re-immersion studies, it appears that a link may exist between the two sound systems. However, it is unclear whether phonetic assimilation will occur from renewed L1 contact or whether both systems will reverse back toward the baseline an equal amount. Given the limited number of longitudinal L1 re-immersion phonetic studies, the question of how, and indeed whether, the two sound systems of language learners reorganize upon returning to the native language environment merits closer inspection.

## 3 The present study

This research analyzes the English and French vowel spaces of 42 English learners of French (ELoF) at the start and end of an RA in a French-speaking country, with a subsection of the participants also recorded 10 months after returning home. Our first research question addresses whether phonetic drift and L2 phonetic development occur over the RA. In the present study, we limit our focus uniquely to systemic change in both languages.

**RQ1:** Does a systemic phonetic change in the learners’ L1 English and L2 French vowels occur from pre-RA to post-RA*?*

Previous comparisons of English and French vowel acoustics generally suggest that the English vowel space is lower than the French vowel space (Lang & Davidson, 2019; Levy, 2009; Levy & Strange, 2008). However, no consistent systemic differences along the front-back cline are commonly observed. As such, if the global formants of French are approximated (Chang, 2012, 2019a; Kartushina & Martin, 2019; Lang & Davidson, 2019), the F1 of the learners’ English vowels is predicted to decrease (systemic raising will occur), and no change along the F2 dimension is expected. However, given that these are not ab initio French learners, the predicted change in ELoF’s L2 vowel system is likely to depend on the quality of their productions at the pre-RA time point. Indeed, if ELoF’s L2 French vowels initially overlap those of native French speakers (NFS), little change is expected, but if they are initially distinct from the native French control group, systemic phonetic change toward the formant values of the target French vowel space is predicted (e.g., Kartushina & Martin, 2019). If a phonetic change occurs in both ELoF’s L1 and L2 vowel systems, our second research question analyzes the persistence of these changes after the learners return home.

**RQ2:** If ELoF’s L1 English and L2 French vowels exhibit systemic phonetic changes over the RA, does L1 re-immersion reverse these changes fully?

Consistent with previous research (e.g., Kartushina & Martin, 2019), any phonetic drift in the L1 English vowel space is predicted to be reversed after 10 months of L1 re-immersion, demonstrating the transient nature of phonetic drift (Chang, 2019b). However, not all research has shown phonetic drift to fully revert, suggesting there may be a degree of variability. Indeed, Chang (2019a) reports sustained drift in the native speech of L1 English participants after the end of their intense course in Korean, as is discussed later in this manuscript. Concerning learners’ L2 phonetic inventory, changes are also likely to reverse upon L1 re-immersion due to reduced L2 input (e.g., Kartushina & Martin, 2019; Sancier & Fowler, 1997; Tobin et al., 2017), although again, not all research has shown reversal of L2 phonetic gains. For example, Engstler (2012) reports that 9 months after a group of L1 English speakers return from studying abroad in France, the phonetic quality of their productions of a variety of L2 sounds is retained. To explore the link between phonetic change in the ELoF’s English and French vowels over time our third research question focuses on variation at the individual level.

**RQ3:** Does interspeaker variation support a relationship between L2 phonetic changes and L1 phonetic drift in the L2 environment and upon L1 re-immersion?

Previous phonetic drift studies analyzing individual variation suggest that speakers who acquire L2 phonetic detail most successfully are also most likely to exhibit phonetic drift in their L1 English vowels in the same direction (e.g., Kartushina et al., 2016). This appears to indicate a relationship between the phonetic change of L1 and L2.

Furthermore, if there is a relationship between the reversal of phonetic drift and loss of L2 gains, we hypothesize that individuals whose L2 gains are reversed upon L1 re-immersion are also the participants who exhibit most return phonetic drift in their L1. Nevertheless, previous research investigating this relationship has not reached conclusive results. For example, Kartushina and Martin (2019) report a significant correlation if all their participants are included but not after excluding a potential outlier. Finally, to address theories of assimilation and dissimilation, our fourth research question analyzes whether ELoF’s L1 and L2 vowels reorganize over the RA and upon L1 re-immersion.

**RQ4:** Does the distance between ELoF’s L1 English and L2 French vowel systems increase, decrease, or remain constant over time?

Given the limited evidence for phonetic category dissimilation in the earlier literature review of longitudinal phonetic drift studies, the present research hypothesizes that if a substantial change occurs in the learners’ L2 French vowel productions over the RA, the distance between the L1 English and L2 French vowel spaces remains constant (e.g., Chang, 2012, 2019a; Kartushina & Martin, 2019; Sancier & Fowler, 1997). If, however, little change in the learners’ L2 French vowel inventory is observed from the pre-RA session to the post-RA session, the distance between the L1 English and L2 French vowels is predicted to diminish, consistent with the fundamentals of *phonetic category assimilation* (Flege, 1995, 2007; Flege & Bohn, 2021).

Dispersion between the two systems is also not expected after L1 re-immersion given the lack of evidence for such a process in studies of phonetic drift involving returnees. Phonetic category assimilation appears to be one of the most common outcomes of renewed L1 contact, whereby the distance between the speakers’ L1 and the L2 productions diminishes (e.g., Sancier & Fowler, 1997; Tobin et al., 2017). In one of the only systemic vowel L1 re-immersion studies to date, Kartushina and Martin (2019) report that participants’ native and foreign sounds both reverse toward the pre-test, but it is unclear whether the distance between the two systems actually diminishes. As such, we hypothesize that both the English and French vowel systems will reverse toward the pre-RA baseline, but we make no explicit predictions for whether the distance between L1 and L2 will either stay constant or reduce.

## 4 Methodology

### 4.1 Participants and design

Forty-three L1 ELoF undertook an RA in a French-speaking country. Data from one participant were excluded because they did not return at the post-RA time point, leaving a total of 42 English-speaking learners of French (28 females, 13 males, 1 nonbinary; mean age: 20.5 years old, age range: 19–23). Although 22 of these participants were recorded before their departure (in England), the other 20 were recorded at the start of their RA in Lyon, France. These groups patterned similarly across time points and so were collapsed into one experimental group (*n* = 42). The majority of residences abroad lasted ~6 months and predominantly took place in France (see Table B1, Appendix B, for further information concerning the length of stays and other participant background information). The second data collection session, referred to as the “post-RA” time point, was conducted at the end or just after participants’ stay in the French-speaking country. Subsequently, 27 of the 42 participants returned for a third data collection session, after being re-immersed in the L1 English environment for approximately 10 months. A further three participants also volunteered for the third session but were excluded from this L1 re-immersion time point as they continued to reside in the French-speaking country for a further 10 months after the post-RA session, rather than returning to the L1 English environment like the other participants.

Background information revealed that the 42 participants were generally advanced, instructed learners but had experienced little naturalistic French input before the RA (again see Table B1, Appendix B, for both participants’ LEXTALE [Brysbaert, 2013] performance as an index of French proficiency and the number of years they declared for the length of learning French). There was some heterogeneity at the level of L1 English variety, but individual differences such as these were not deemed grounds for exclusion given that the same participants were recorded longitudinally. None of the data collection sessions lasted longer than 1.5 hrs and participants were compensated for their time with a small monetary token.

Finally, a sample of 10 NFS (6 females, 4 males, mean age: 22.4 years, range: 19–27 years) was included as a reference for the direction of any phonetic change in the learners’ L1 English or L2 French production of vowels. The majority of these participants were from the Parisian districts of France although two were also from Belgium, and the group were within their first month of an RA in the United Kingdom.^2^

### 4.2 Procedure and stimuli

The participants in the experimental group completed production tasks in both English and French, along with a French perception task. For the purposes of space in the present study, only the production tasks are reported here. A short video was watched in the language of the subsequent tasks to encourage learners to enter a more monolingual language mode (Grosjean, 1998) and for the experimental group, the English tasks were always completed first to reduce L2 priming effects. The NFS only performed the French tasks.

The stimulus phrases used to elicit productions contained one lexical item of interest, featuring one of 10 English monophthong vowels or one of 10 French monophthong vowels. Six different items for each vowel were uttered per speaker with the order of phrases pseudorandomized using *Experiment Builder* (SR Research, 2011) such that no two items of the same vowel were consecutive. The phrases, items, and vowels are almost identical to those used in Lang and Davidson (2019) (see Appendix A).^3^ As such, tokens are in CVC (consonant–vowel–consonant) contexts, with the vowels surrounded by either stops or fricatives to facilitate segmentation. Although the following consonant was sometimes situated across a word-boundary due to cross-linguistic lexical constraints, lexical items were never in the phrase-initial or phrase-final position, again consistent with the original design by Lang and Davidson (2019). To distinguish between languages in data visualization, the English vowels were labeled using lexical sets (Wells, 1982) and IPA symbols were reserved for French vowels.

At the first time point, recordings were made in person and in a quiet location, with individual phrases presented to participants using *Experiment Builder* (SR Research, 2011) and read aloud at a conversational pace. Due to COVID-19, at the second and third time points participants recorded themselves in a quiet location and, once more, read the isolated phrases presented on their computer screen. At the second and third data collection time points, the author video-called the participants throughout the session to verify that the tasks were being completed in one stint, in a similar fashion to the pre-RA test. At the first time point, a Sennheiser e845S dynamic microphone was used, whereas in Sessions 2 and 3, participants used the best microphone available to them.^4^

At all time points, participants also answered an online questionnaire featuring a language background and engagement section based on the tool developed by Mitchell et al. (2017). Although these data are not analyzed statistically in the present study, French input appears to increase and English input decreases from pre-RA to post-RA, as expected. Furthermore, after 10 months’ L1 re-immersion, French input levels dramatically reduce and the ratio of English to French input resembles that of the initial pre-RA time point. In this questionnaire, participants were also asked to select words from a long list of L2 stimuli if they were unfamiliar with the words’ pronunciation due to rarely coming across them in their learning. These tokens were subsequently excluded from analysis (*n* = 124, 0.01% of the overall data).

### 4.3 Data analysis

All recordings were made at a sample rate of at least 44.1 kHz with the audio bit depth set at 16-bit, or downsampled to this quality to ensure consistency between recordings. An orthographic, time-stamped transcription of recordings was obtained using a script written by the author in *Praat* (Boersma & Weenink, 2020). The script used the silences between phrases in the recording to determine the start and end of each utterance, and time-stamps were inserted at these positions. The phases were then labeled in the order in which the stimuli were presented to the participant, and the labeling was checked by hand in the final stage of the script. This transcription was subsequently forced aligned using *SPPAS* (Bigi & Meunier, 2018), a software that is compatible with both English and French speech data. The segmentation of all vowels of interest was hand-checked and adjusted where necessary, once more using a *Praat* script. The onset and offset of the vowel were labeled as the first and last reliable glottal pulses for which F1 and F2 were also visible in the spectrogram (see Chang, 2019a, for a similar method).

Once the files were prepared as detailed above, each participant’s mean F0 was calculated across all phrases and formant extraction was subsequently automated in *Praat* using the standard Burg LPC settings (Childers, 1979) with the window length set at 25 ms, five specified as the maximum number of formants, and a pre-emphasis of 50 Hz. The maximum formant frequency was set at 5000 Hz for lower voices (mean F0 < 164.5 Hz) and 5500 Hz for higher voices (mean F0 > 164.5 Hz).^5^ Formant readings were taken from the midpoint of each vowel and outliers were determined by scaling F1 and F2 by vowel: *z*-scores lower than minus 3 or higher than positive 3 were removed (*n* = 262, 1.87% of overall data). This has been used as a standard method of labeling outliers and reducing formant tracking errors within bilingual speech samples (Kirkham & McCarthy, 2021). The remaining data were normalized using a variation of the Nearey 2 (Nearey, 1978) method to reduce interspeaker variation based primarily on vocal tract size. Nearey 2 is a vowel extrinsic method which involves subtracting the mean log of Formants 1 to 3 across all vowels from the log of each formant reading. The method employed by the present study, outlined more fully in Barreda and Nearey (2018), is identical to Nearey 2, other than the fact that it uses a regression model output estimate of the log mean rather than the sample log mean. Barreda and Nearey (2018) report that this method reduces the biases arising from missing data for different speakers. As such, it is less likely to “overnormalize” the present data and is less sensitive to the exclusion of unfamiliar items and/or outliers.

### 4.4 Statistical models

Linear mixed effects models (LMERs) were fitted using the *lme4* (Bates et al., 2015) and *lmerTest* (Kuznetsova et al., 2020) packages in *R* (R Core Team, 2018). The first two models determined whether there were any significant changes between ELoF’s L1 English vowel system at the pre-RA time point and the native French vowel space of the control group in terms of both F1 (Model 1) and F2 (Model 2). In both models, the variable Group (effects coded 3 ways: NFS [reference], ELoF L1 English pre-RA, ELoF L2 French pre-RA) was fitted as a fixed effect.

Two further models were fitted to analyze the change in ELoF’s in L1 English and L2 French vowels across the three time points along both F1 (Model 3) and F2 (Model 4). These models were fitted with an interaction between time point (effects coded: post-RA [reference], pre-RA, L1 re-immersion) and language (dummy coded: English, French). Finally, Model 5 analyzed the difference between each participant’s mean F1 in English and French. Time point (effects coded: post-RA [reference], pre-RA, L1 re-immersion) was fitted as a fixed effect to determine whether ELoF’s L1–L2 distance changed over time.

In Models 1–4, random intercepts of both Speaker and Word were included (Baayen et al., 2008; Linck & Cunnings, 2015), and in preliminary models, the random structure was kept maximal by including by-Speaker and by-Word random slopes, but only for variables that were manipulated within Speaker and within Word (Barr et al., 2013).^6^ All models were backward reduced using the Step function from the *lmerTest* package (Kuznetsova et al., 2020), a function that calculates likelihood ratio tests for each part of the random structure (using *ranova*) and *F*-tests for the fixed effects (using *drop1*). To determine whether other factors explained phonetic change over time (such as the participant information specified in Table B1, Appendix B), these were added to Models 3 and 4 as an interaction with Time Point. Such variables included length of learning French (years); French proficiency (Lextale); length of RA (months); length of L1 re-immersion (months); reported L1 English variety (South England, the United States, Midlands England, Other [Scotland/North England]); previous naturalistic French exposure (some vs. none); whether French was participants’ only foreign language (yes vs. no); and, finally, type of RA (English teacher/assistant vs. university student).^7^ However, according to *F* statistics, none of these interactions were significant and so they were not controlled for in final models. Where “pairwise comparisons” are reported in the text, these refer to Tukey-corrected estimated marginal mean comparisons conducted post hoc using the Kenward–Roger degrees-of-freedom method (Kenward & Roger, 1997) in the package *Emmeans* (Lenth et al., 2020). All five final models as well as their respective *R* code can be found in Appendix C and are openly available online: https://osf.io/jszw3/.

## 5 Results

### 5.1 Baseline results (Pre-RA)

**Figure 1.**
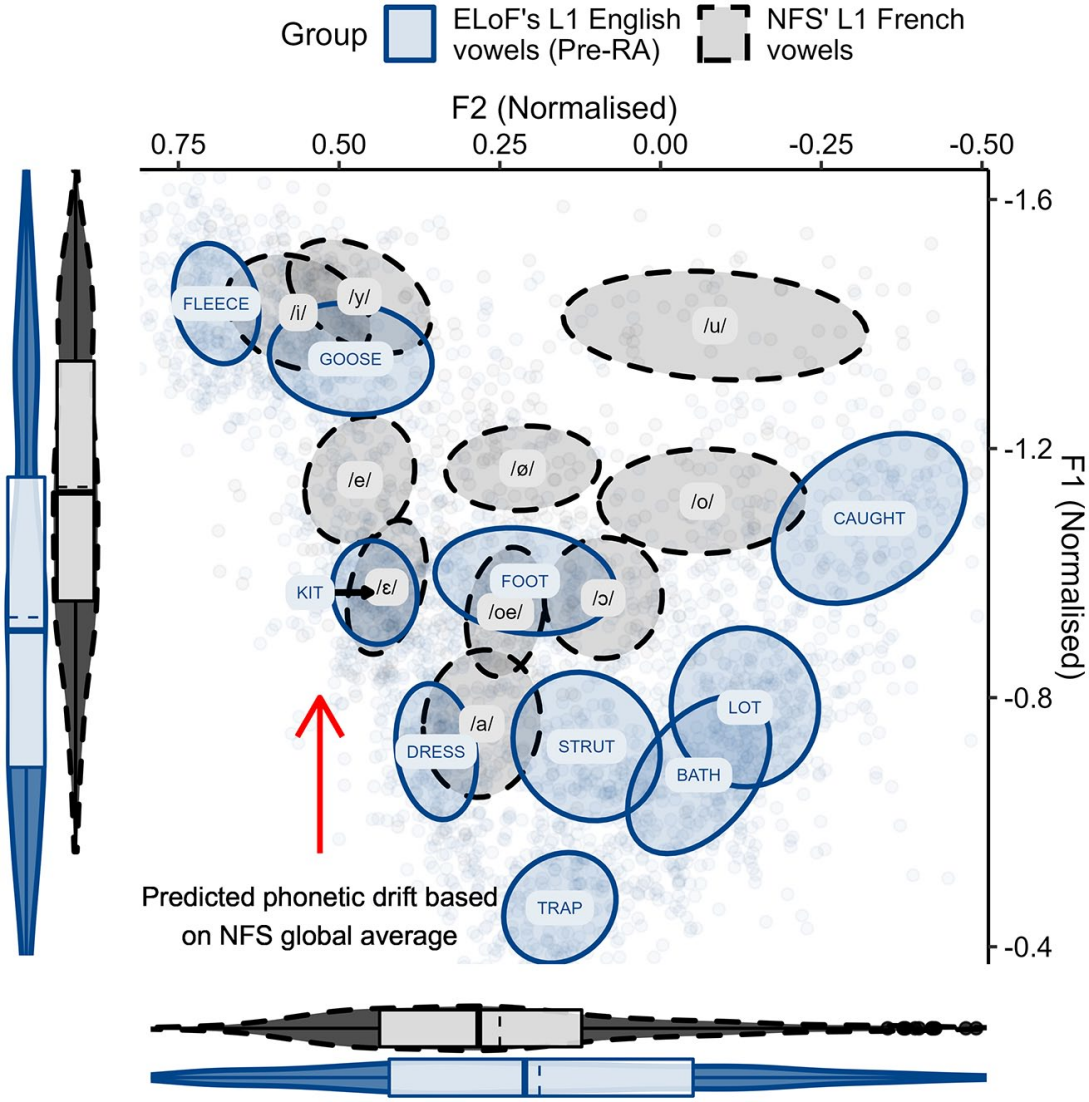
ELoF’s native English vowels (blue, solid) before the residence abroad compared with the French vowel space of the native speakers (black, dashed). Plot demonstrates that ELoF’s L1 English vowels are lower than those of NFS but similar in terms of F2. Note that as an indication of by-segment variation, ellipses show 35% confidence intervals around the mean (plotted centrally as text) and boxplots demonstrate global distribution along the axes (dashed line = mean, solid line = median).^a^ ^a^These parameters also apply to Figures 2 [Fig fig3-00238309221133100][Fig fig4-00238309221133100][Fig fig5-00238309221133100]–6.

**Figure 2.**
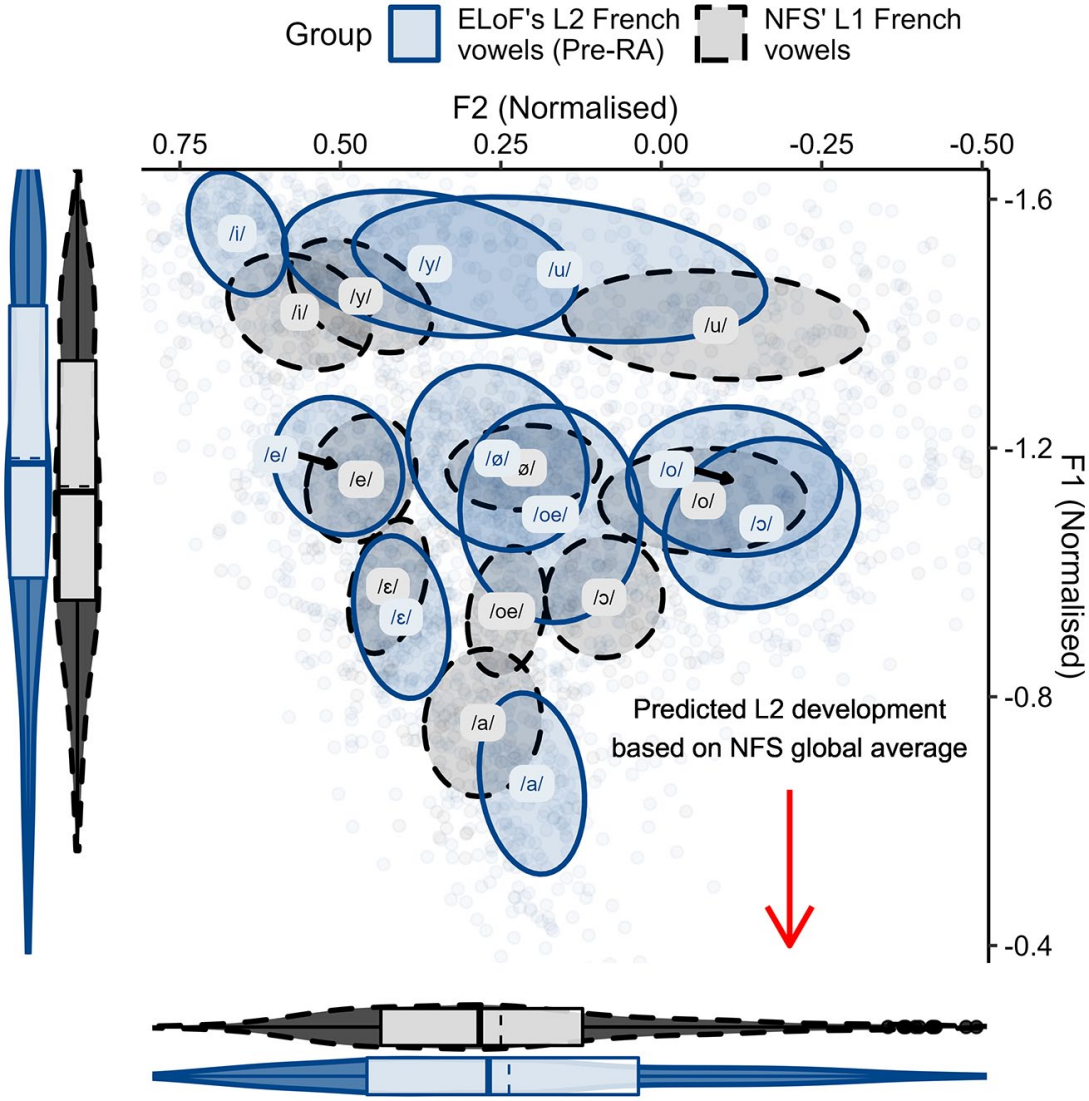
ELoF’s L2 French vowels (blue, solid) before the residence abroad compared with the native French vowel space (black, dashed). Plot demonstrates that ELoF’s L2 French vowels are slightly higher than those of NFS but similar in terms of F2.

The first analysis compared the French vowels of the NFS group and both ELoF’s native English (Figure 1) and ELoF’s L2 French vowels (Figure 2) at the first time point (pre-RA).

A visual comparison between languages in Figure 1 suggested that the native English vowels were lower than those of native French at the pre-RA time point, and this was confirmed by Model 1 (β = 0.22, *SE* = 0.05, *t* = 4.02, *p* < .001). As such, we predicted that L1 drift would be upward over the RA if the learners’ English vowels shift toward the global formant values of native French. In contrast, the French comparison of Figure 2 indicated that although some of ELoF’s L2 French vowels were higher than the targets (especially, the high vowels) and some were lower (namely, /a/), on average ELoF’s L2 system was slightly higher at the pre-RA time point compared with the global values of the NFS vowel space (see *y*-axis marginal box plot of Figure 2). This was also confirmed by Model 1 (β = −0.05, *SE* = 0.01, *t* = −3.06, *p* < .01). As such, systemic lowering in ELoF’s L2 French was expected over the RA if their vowels approximate the global formant values of the foreign language.

The second formant was also analyzed but no effect of Group was observed: there was little difference between the average frontness of NFS’ vowels and both ELoF’s English vowels (β = −0.07, *SE* = 0.05, *t* = −1.30, *p* = .20) and ELoF’s French vowels (β = −0.02, *SE* = 0.01, *t* = −1.46, *p* = .15). No phonetic change over time was therefore expected along the front-back cline in either of ELoF’s languages.

### 5.2 Results for phonetic drift and L2 development over the RA

**Figure 3. fig3-00238309221133100:**
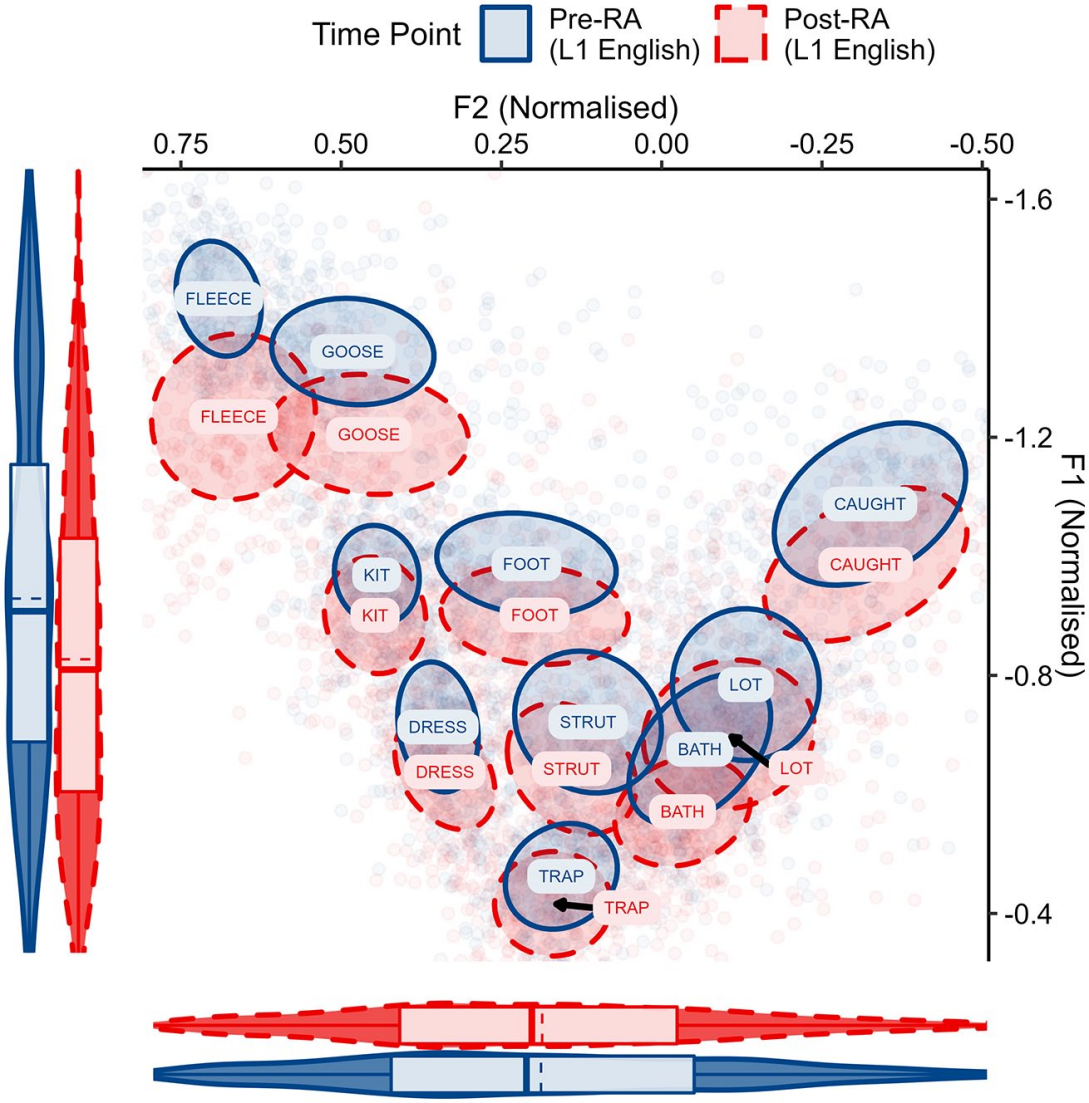
ELoF’s L1 English vowels before the residence abroad (blue, solid) and afterward, at the post-RA time point (red, dashed). Plot demonstrates that ELoF’s L1 English vowel system lowers but does not change along F2.

First, a descriptive plot of ELoF’s L1 English vowels was created to observe any change between the pre-RA and post-RA sessions (Figure 3).

**Figure 4. fig4-00238309221133100:**
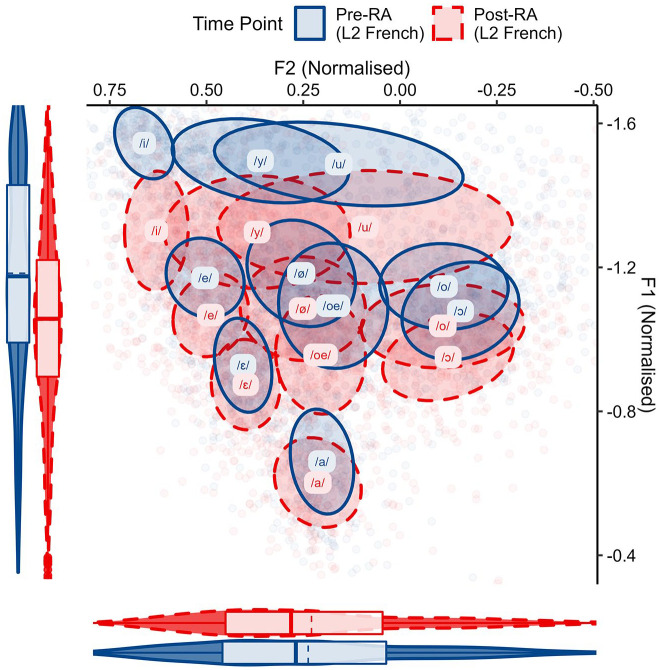
ELoF’s L2 French vowels at the pre-RA time point (blue, solid) and at the post-RA time point (red, dashed). Plot demonstrates that ELoF’s L2 French vowel system lowers but does not change along F2.

This indicated that instead of ELoF’s L1 English vowel space raising as expected, what occurred was in fact the opposite: the learners’ English vowels lower systemically. Nevertheless, this drift is in the same direction as their L2 phonetic development. The change in ELoF’s L2 French vowels can be seen in Figure 4.

This indicated that the L2 French vowel system lowers substantially over the RA, bringing it into line with the native French global F1 values.

The systemic lowering in both vowel spaces was confirmed by statistical analyses. Model 3 analyzed the change in F1 and the fitted interaction between time point and language not only revealed that ELoF’s L1 English vowels lowered significantly from the pre-RA to post-RA (β = 0.10, *SE* = 0.01, *t* = 9.73, *p* < .001), but also that ELoF’s L2 French vowels lowered even more than their L1 English vowel space (β = 0.03, *SE* = 0.01, *t* = 2.56, *p* < .05). The second formant was also analyzed, but ELoF’s English vowels did not change over the RA (β = −0.002, *SE* = 0.01, *t* = −0.30, *p* = .76) and their French vowels were no different (β = −0.01, *SE* = 0.01, *t* = −0.84, *p* = .40).

### 5.3 Persistence of phonetic drift and L2 gains upon L1 re-immersion

**Figure 5. fig5-00238309221133100:**
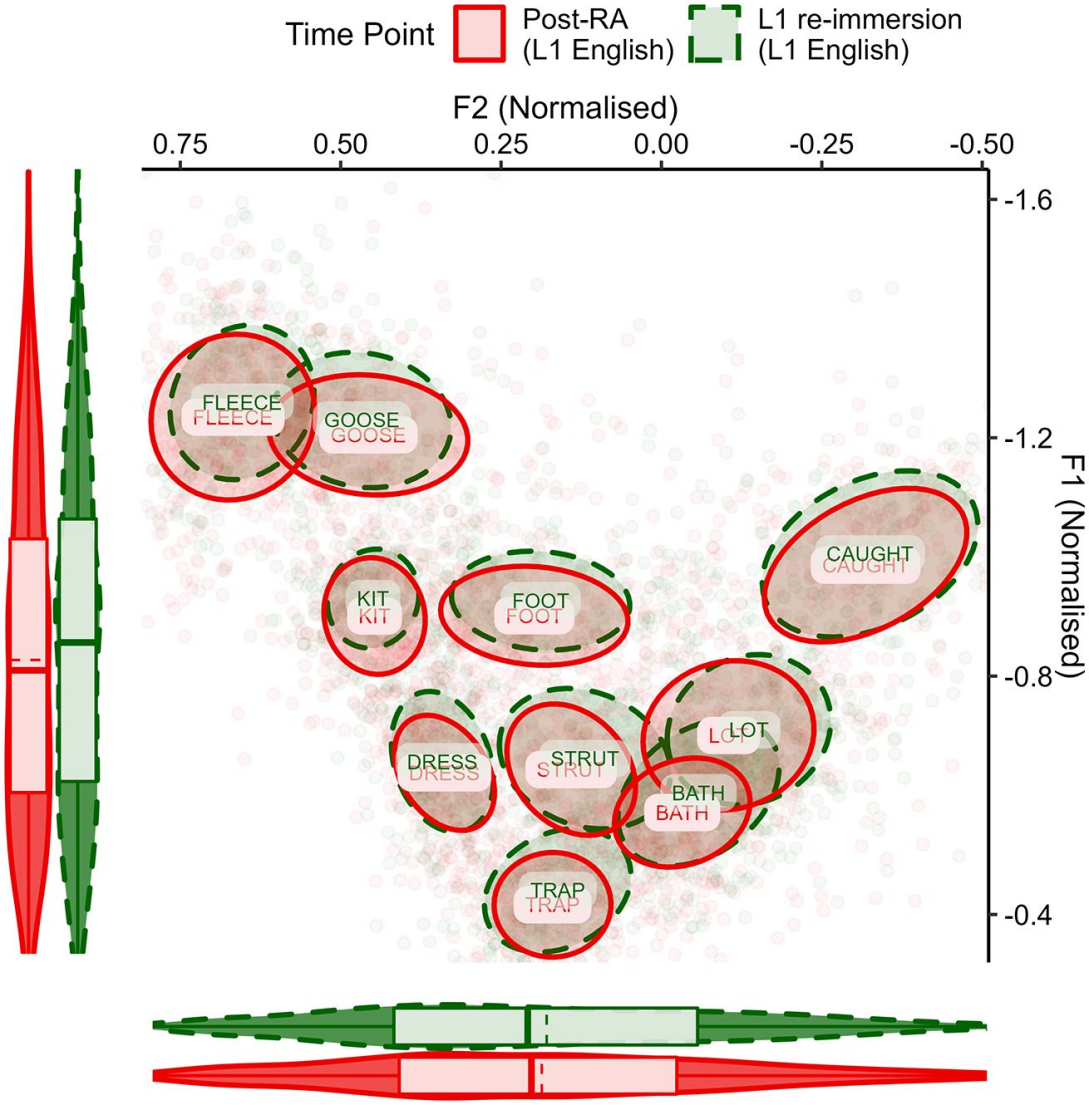
ELoF’s L1 English vowels at the post-RA time point (red, solid) and the L1 re-immersion time point (green, dashed). Plot demonstrates that ELoF’s English vowel system raises slightly but does not change along F2.

Next, the change in ELoF’s vowels between the post-RA session and the 10-month L1 re-immersion time point was analyzed. ELoF’s English vowel data for these sessions were first compared visually in Figure 5.

From this graph, ELoF’s L1 English vowels appear to exhibit slight raising between the post-RA and the L1 re-immersion time points, and Model 3 confirmed that this was significant (β = −0.03, *SE* = 0.01, *t* = −2.56, *p* < .05). Nevertheless, pairwise comparisons suggested that ELoF’s L1 English vowels remain substantially lower at the re-immersion time point compared with the pre-RA session (β = 0.07, *SE* = 0.01, *t* = 6.53, *p* < .001). As such, L1 was not entirely reset to its value at the baseline.

**Figure 6.**
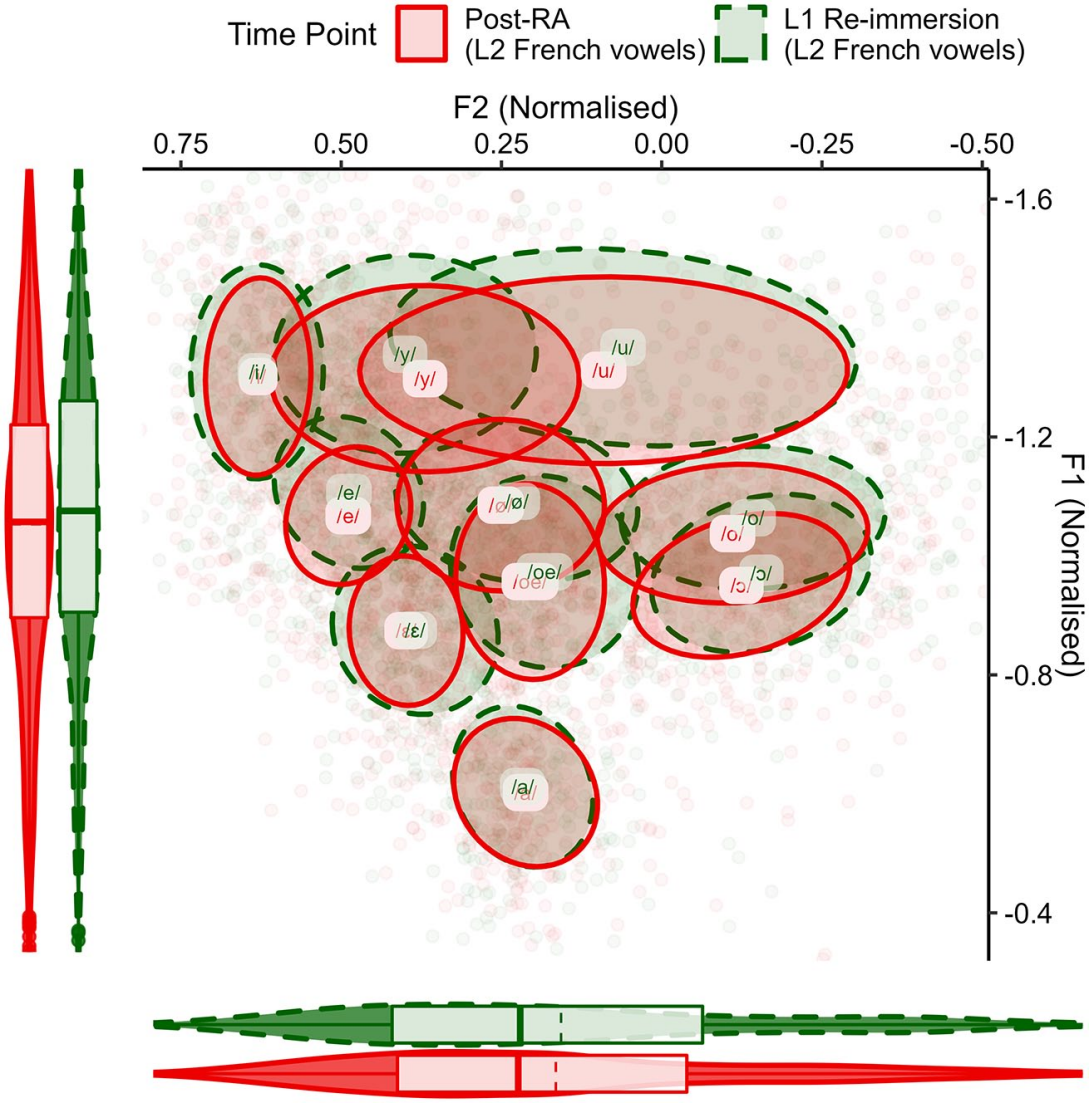
ELoF’s L2 French vowels at the post-RA time point (red, solid) and the L1 re-immersion time point (green, dashed). Plot suggests no systemic change along F1 or F2.

In contrast, ELoF’s L2 French vowel space did not appear to exhibit raising to the same extent from the post-RA session to the L1 re-immersion time point. This is demonstrated in Figure 6.

Pairwise comparisons within the Time Point × Language interaction of Model 3 suggest that ELoF’s French vowels did not significantly shift upward after L1 re-immersion (β = −0.02, *SE* = 0.02, *t* = −1.18, *p* = .84), and the vowels remained substantially lower than at the pre-RA time point (β = 0.12, *SE* = 0.02, *t* = 6.48, *p* < .001). These results indicate that although there was a slight reversal of the phonetic drift after returning home in ELoF’s L1 English, limited loss of the L2 gains appears to have occurred in the learners’ French vowels.

F2 was analyzed once more in Model 4, but no significant differences between the post-RA and L1 re-immersion time points were observed in English (β = −0.01, *SE* = 0.01, *t* = −1.58, *p* = .12), and the change in French was not significantly different from the English results (β = −0.001, *SE* = 0.01, *t* = −0.13, *p* = .90).

### 5.4 Interspeaker variation

**Figure 7.**
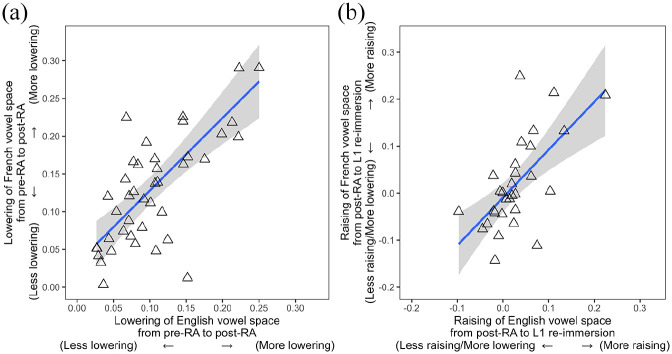
(a) Correlation between phonetic drift over the RA (English vowel lowering) and L2 development (French vowel lowering) for all 42 participants. (b) Correlation between reversal of phonetic drift after L1 re-immersion (English vowel raising) and reversal of L2 gains (French vowel raising) for the 27 returnees.

Subsequently, we analyzed the link between L1 and L2 phonetic change at an individual level by running Spearman’s rank correlations. The amount of lowering over the RA in ELoF’s English and French was calculated as follows: post-RA F1 minus pre-RA F1, and the amount of raising after the RA was calculated as follows: −1 × (L1 re-immersion F1 − post-RA F1). The latter calculation ensured the values for “raising” were all positive, facilitating interpretation. The first correlation (*n* = 42) revealed that over the RA, individuals making the most development (in terms of systemic vowel lowering) in their L2 French were also those who exhibited most substantial phonetic drift (vowel lowering) in their L1 English (*rho* = 0.66, *p* < .001), reaffirming the likelihood of a link between these two systems. A second correlation (*n* = 27) revealed that individuals whose L2 French vowels raised the most from the post-RA session to the L1 re-immersion time point were also those whose L1 phonetic drift reversed most from the post-RA session to the L1 re-immersion session (*rho* = 0.64, *p* < .001), indicating that the reversal of phonetic drift may be linked to loss of L2 gains. Both of these correlations are plotted in Figure 7.

### 5.5 Assimilatory or dissimilatory drift?

**Figure 8.**
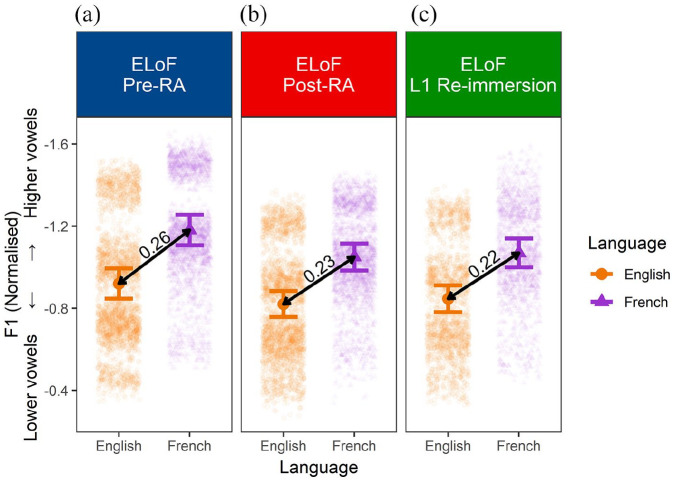
(a, blue) F1 estimates for ELoF’s L1 English (orange, circle) and L2 French vowels (purple, triangle) at the pre-RA time point. (b, red) F1 estimates for ELoF’s L1 English (orange, circle) and L2 French vowels (purple, triangle) at the post-RA time point. (c, green) F1 estimates of ELoF’s L1 English (orange, circle) and L2 French vowels (purple, triangle) at the L1 re-immersion time point. Plot demonstrates that the distance between L1 and L2 productions decreases over the RA. Note that error bars represent 95% confidence intervals.

Finally, to analyze how the distance between the height of ELoF’s L1 English and L2 French vowels changes over time, the fitted interaction between time point and language in Model 3 was plotted and the difference between the estimate for each language at all three time points was calculated (see Figure 8).

This revealed that the distance between learners’ L1 English and L2 French vowels diminishes and that this change was most substantial between the pre-RA test and the post-RA test. Model 5 was fitted to confirm that the distance between each participant’s English mean F1 and French mean F1 reduced significantly from the pre-RA session to the post-RA session (β = −0.03, *SE* = 0.01, *t* = −2.61, *p* < .05). At the same time, this model suggested that the change from the post-RA to the L1 re-immersion time point did not reach significance (β = −0.01, *SE* = 0.01, *t* = −0.57, *p* = .57).

## 6 Discussion

This research analyzes the L1 English and L2 French vowel spaces of 42 L1 ELoF, before and after they spend a 6-month RA in a French-speaking country, as well as 10 months after a subsection of the participants (*n* = 27) returns to the L1 English environment. In this section, we return to the research questions stated in Section 3 and evaluate the respective hypotheses in turn.

Our first line of investigation **(RQ1)** determined whether the acoustic properties of learners’ L1 English and L2 French vowels change over the RA. Baseline results for F1 suggested that the native French vowel space was higher than that of English at the pre-RA time point predicting that the learners’ English vowel system would become more raised over the RA. In contrast, the baseline results for ELoF’s L2 French vowels suggested that they were on average slightly higher than the native French vowels at the pre-RA session, predicting that the L2 vowel space would lower to approximate L2 norms over the RA. No change was expected along F2 for either of ELoF’s languages because no differences were observed between the advancement of the native French vowel space and that of both ELoF’s L1 English and L2 French vowels.

Statistical analyses revealed systemic phonetic drift in the learners’ English vowel space, but not in the predicted direction: the vowel space lowered, drifting away from the norms of the L2 French input. However, as expected, the learners’ French vowels also lowered significantly in the direction of native French norms and no systemic horizontal shift in either vowel space was observed. These results indicate a relationship between acquisition of L2 phonetic detail and systemic L1 phonetic drift. Indeed, the phonetic change in the learners’ L2 French vowel space is entirely consistent with the change in the L1 English vowels. That is, although the L1 English vowels could have drifted upward to approximate the global formant values of the L2 French input (Chang, 2012, 2019a), they lower in tandem with the phonetic changes occurring in L2.

Before elaborating on the RA results, it is important to consider why these learners exhibit a higher L2 French vowel space at the pre-RA time point compared with the NFS vowels, despite the learners’ English vowels being lower than in native French. Indeed, it is not the first time that experienced L1 ELoF have been shown to produce French vowels more raised than those of NFS, especially in the upper regions of the vowel space (e.g., Flege, 1987; Flege & Hillenbrand, 1984; Lang, 2015; Lang & Davidson, 2019). Although it is only possible to speculate given the lack of any data preceding the pre-RA time point in the present study, it is likely that ELoF attempt to minimize overlap between their L1 English and L2 French vowels. That is, ELoF’s L2 French vowels are prevented from converging on the height of the native French vowel space because this may situate the learners’ L1 and L2 too close together, leading the L2 vowels to be forced upward and resulting in the L2 target being overshot. Indeed, bilingual speakers strive to keep their two systems distinct (Guion, 2003), which is equally a probable explanation for tandem drift: the L2 French vowel space lowers over the RA, putting pressure on the L1 English vowel space to also lower or risk overlap with the L2 French vowel space.

Chang (2012) notes a similar case of L1 and L2 phonetic detail overshooting L2 norms. In that study, the L1 English speakers who begin learning Korean produce aspirated Korean plosives at first with shorter VOTs than the L2 Korean norms, presumably due to the influence of English which has relatively shorter VOT for fortis stops. The participants’ L2 VOTs lengthen as their experience of Korean increases, quickly reaching the Korean norms by week 2 of the course. However, from weeks 2 to 5, the VOT lengthening continued in their L2, meaning the learners ended up “overaspirating” the Korean plosives (Chang, 2012, p. 259). Importantly, the VOT of the learners’ English stops also continues to shift well past the norms of the Korean input, suggesting that the L1 drifts in tandem with L2 phonetic development rather than converging on the values found in the input.

This was reemphasized by the findings in Chang (2019a). Although cross-linguistic comparisons suggest that the vowel space of Korean is generally more retracted (globally lower F2) than in English (see Chang, 2012, p. 255), Chang (2019a) highlights that the native English vowel space of the L2 Korean learners becomes more fronted between Weeks 1 to 52 of learning Korean. As such this drift is away from the norms of Korean. Although this fronting is unlikely to be predicted by the norms of the L2 input, the learners’ L2 Korean vowel space also appears to show fronting over time, indicating that the L1 change is likely linked to fluctuations in the trajectory of L2 phonetic development.

Overall, these results indicate that drift cannot always be accurately predicted “solely on the basis of cross-linguistic differences between similar sounds; rather, there are other intervening factors” (Chang, 2019b, p. 196). We suggest that L2 phonetic development is of particular importance in this respect. Indeed, if the development of L2 is not also taken into account alongside L1 speech plasticity, phonetic drift studies risk providing an incomplete account of this phenomenon.

Having observed a link between phonetic drift and L2 phonetic development from the pre-RA session to the post-RA session, we next analyzed whether a similar L1–L2 link would be found between the post-RA test and the L1 re-immersion time point, which took place 10 months after participants returned to the L1 English environment **(RQ2)**. Because previous research has suggested that phonetic drift is reversible (Chang, 2019b) and that systemic changes to production of vowels in a foreign language are difficult to maintain upon reduced L2 input (Kartushina & Martin, 2019), we predicted that all systemic changes occurring over the RA would be fully reversed in both languages upon returning home to the L1 English environment.

Results partially support this hypothesis. In terms of the first formant, for example, a slight reversal in the phonetic drift of ELoF’s English vowel space was observed between the post-RA session and the L1 re-immersion time point, as demonstrated by a global shift upward in the vowel space. In contrast to our hypothesis, however, ELoF’s L1 English vowels remained significantly lower at the L1 re-immersion time point than at the pre-RA baseline session. Although the transience of phonetic drift (Chang, 2019b) is therefore demonstrated by this partial reversal, the acoustic quality of the L1 vowel system is not entirely reset upon L1 re-immersion (c.f. Kartushina & Martin, 2019). Furthermore, analyses of ELoF’s L2 French vowels suggested that the vowel-lowering observed over the RA was not reversed from the post-RA session to the L1 re-immersion time point. Results align, instead, with previous research that has reported a certain degree of retention in the phonetic quality of foreign language learners’ L2 productions within a year of returning home (Engstler, 2012).

One possibility is that the native and foreign vowel inventories in Kartushina and Martin (2019) are more susceptible to the effects of L1 re-immersion because the phonetic changes are less well ingrained than in the present study. Indeed, those learners were immersed in the foreign language environment for a shorter amount of time (2 weeks in that study vs. 6 months here) and the length of L1 re-immersion was also almost nine times the length of the stay abroad, whereas the length of L1 re-immersion in the present study is not even twice the length of the average RA length. This is an important factor according to Chang (2012, p. 265) who posits that L1 phonetic reversibility “in the continued absence of L2 exposure will depend greatly on the total amount of L2 experience accrued.” Nevertheless, because Time Point did not interact with the variables length of RA and length of L1 re-immersion in the present study, the importance of *time* itself as a predictor of drift and reverse drift is unclear. Potentially, the amount of change in the relative proportion of L1 versus L2 input may, instead, offer a more fruitful avenue to explore in future research rather than the length of time per se.

An alternative reason for the native sound system not fully resetting could be that sustained phonetic drift is simply not reliant on large amounts of L2 contact, and thus, can be prolonged despite L1 re-immersion. Indeed, even L1 English speakers who rarely use Korean in Chang (2019a) exhibit sustained L1 phonetic drift a year after this L1 change is first observed. However, in that study, participants do in fact remain in the L2 Korean environment, which allows L2 to be processed incidentally (Chang, 2019a, p. 107). Although findings of the language engagement questionnaire (Mitchell et al., 2017) are not explored statistically in this study, descriptive statistics do indicate that a certain level of engagement with French activities continues after returning to the L1 English environment. Although this is a similar level of French engagement to the pre-RA session, this small quantity of French input may still suffice to maintain a substantial amount of the phonetic drift from the RA.

Next, the link between phonetic change in the native and foreign language both over the RA and upon L1 re-immersion was investigated at the individual level in **RQ3)**. The first correlation indicated a relationship between L1 drift and L2 phonetic change: the learners whose L2 French vowel space lowered most over the RA, were also the learners who exhibited the most substantial L1 English vowel lowering (i.e., phonetic drift). Such findings are consistent with previous research focusing on between-speaker variability in phonetic drift. For example, Kartushina et al. (2016) report that the most substantial phonetic driftees for L1 French /o/ were also the participants who exhibit the most development in L2 Danish /ɔ/ production. The present results extend these findings by demonstrating that not only is this L1–L2 relationship present at the individual segment level, but also between systemic properties of language learners’ L1 and L2 vowel productions. We return to this idea later when considering results in relation to the *SLM-r* (Flege & Bohn, 2021).

A second correlation suggested that this relationship persisted upon L1 re-immersion. Indeed, the amount of reverse phonetic drift exhibited in a learner’s L1 English (i.e., systemic English vowel raising) corresponded to the extent to which L2 phonetic development was reversed from the post-RA session to the L1 re-immersion time point (i.e., systemic raising in their L2 French vowels). This finding mirrors that of Kartushina and Martin (2019) who find that the reversal of phonetic drift in L1 Spanish and Basque vowels upon returning home is correlated with the reversal of phonetic gains in English.^8^ This L1–L2 relationship at the individual level may offer a further explanation as to why phonetic drift does not fully reset upon L1 re-immersion at the group level in the present study: unlike the group results of Kartushina and Martin (2019), here L2 gains are not reversed which may prevent a full reset of L1 phonetic drift. Although it is too early to tell whether these patterns at the individual level can be generalized consistently, L2 phonetic development does appear to offer a promising line of enquiry for why such variability in longitudinal phonetic drift exists among language learners (see e.g., Schuhmann & Huffman, 2015).

Although the findings discussed thus far focus on the phonetic changes of each language between time points, our fourth research question **(RQ4)** analyzes the distance between L1 and L2 along a given acoustic dimension. Our predictions were that if L2 phonetic development occurs over the RA (i.e., systemic French vowel lowering) phonetic change in L1 would occur to a similar extent, resulting in a sustained L1–L2 distinction (e.g., Chang, 2012, 2019a; Kartushina & Martin, 2019; Sancier & Fowler, 1997). Upon L1 re-immersion, we also predicted that the reversal of L1 and L2 phonetic changes would occur in both languages, with the cross-linguistic distance remaining relatively constant (e.g., Kartushina & Martin, 2019) or diminishing (e.g., Sancier & Fowler, 1997; Tobin et al., 2017).

Results comparing data from both languages suggest that the distance between learners’ L1 English and L2 French vowel space diminishes over time, and that this change is most apparent between the pre-RA time point and the post-RA time point. Indeed the learners’ L2 French vowel space lowers more than their L1 English over the RA, which leads to a smaller distinction between languages in terms of global F1 values.

Although these assimilatory results are in contrast to our hypothesis that the L1–L2 distance would not change over the RA, they support the notion that tandem drift (significant L1 and L2 phonetic changes in the same direction) and phonetic category assimilation (reduced distinction between L1 and L2) are not mutually exclusive. In *SLM-r* terms, we suggest that tandem drift may not necessarily constitute evidence of new phonetic category formation, but rather that the perceptual link between L1 and L2 vowels has not been “sundered” (Flege & Bohn, 2021, p. 40), leading to assimilatory drift over time. This link does not appear affected by L1 re-immersion given that the L1–L2 distance at the third time point is still much smaller than at the pre-RA session. This mirrors findings of previous L1 re-immersion studies in which L1–L2 assimilation occurs to some degree (e.g., Sancier & Fowler, 1997; Tobin et al., 2017). Although we turn to the implications for the distinction between drift and attrition later, it is worth noting that findings of L1–L2 assimilation also build on recent longitudinal phonetic attrition research. For example, Kornder and Mennen (2021) report that the distinction between the L1 Austrian German and L2 English speech of the actor Arnold Schwarzenegger appears to lessen as the length of time residing in the United States increases. Indeed, over time the acoustic difference between Schwarzenegger’s VOT for voiceless stops in Austrian German and English reduces, as does the acoustic distance between a number of the L1 and L2 vowel categories (Kornder and Mennen, 2021).

Overall, our results are consistent with the *SLM-r* claim that “a merger of the phonetic properties” (Flege & Bohn, 2021, p. 42) of L1 and L2 can occur in the long-run, leading to assimilatory drift. However, it would appear that this link between L1 and L2 vowels exists at a systemic level as suggested by Guion (2003), rather than uniquely at the level of the individual segment. It is unclear how the *SLM-r* can accommodate such findings given that its assumptions are rooted in the perceptual association of L1 and L2 position-sensitive allophones (Flege & Bohn, 2021, p. 64). Instead, Chang (2012, p. 255) suggests that “cross-language linkages” are developed not only for individual segments but also phonological classes and global properties of sound systems. As such, composite L1–L2 representations such as the like proposed in the *SLM-r* may yet exist, but apply at multiple levels. If “systemic” representations are phonetic in nature, which appears a reasonable assumption (see e.g., Mennen et al., 2010 for research into language-based phonetic settings), language learners face two phonetic learning challenges to acquire acoustic and articulatory detail in L2: one which involves learning the distributions associated with individual segments, and one which involves internalizing the distributions of the properties associated with the system as a whole. Although the present research limits its scope to the latter of these, we do not deny that variation at the level of the segment exists, and future research would do well to explore the relationship between these segmental and systemic learning processes.

To conclude this discussion, we consider briefly the broader implications of our results for the phenomena of *phonetic drift* and *phonetic attrition*. Indeed, this study revealed changes to L1 acoustic properties which show at least some sign of reversal upon renewed L1 contact, a finding consistent with the assumed reversibility of phonetic drift (Chang, 2019b). According to Chang (2019b), phonetic attrition is less likely to undergo such a reversal. However, we also found that the quality of ELoF’s English vowels was not entirely reset to the values observed at the pre-RA baseline (before extensive L2 exposure). As such, these findings indicate that over time, fluctuations in L1 phonetic detail (i.e., phonetic drift), may also constitute a gradual decrease in phonetic similarity to monolingual speakers of L1 (phonetic attrition). This was first proposed in Chang (2019b, p. 203) but had yet to be supported by longitudinal data such as those of the present study. Nevertheless, in the present study, no comparison was made between ELoF at the pre-RA time point and a group of native English monolinguals to confirm that they had not already undergone phonetic attrition in some respect by the first data collection session. Future research may therefore benefit from an L1 monolingual control as well as tracking several instances of phonetic drift*—*and its reversal*—*longitudinally. Indeed, it is only through observing several instances of L1/L2 phonetic plasticity that we might determine whether the rolling average of these fluctuations constitutes gradual divergence from L1 monolinguals and, therefore, attrition.

## 7 Concluding remarks

This research analyzed the L1 English and L2 French vowel spaces of L1 ELoF over a 6-month RA in a French-speaking country and 10 months after returning home. Our primary objectives were (a) to determine whether L1 phonetic drift is linked to L2 phonetic development, (b) to analyze whether L1 and L2 phonetic changes over an RA were reversed upon extensive L1 re-immersion, and (c) to establish whether the distance between L1 and L2 vowel systems varies or remains constant both in the L2 environment and upon L1 re-immersion. Results revealed an apparent link between the phonetic drift in the learners’ L1 English vowels and the development of their L2 French vowel production. Concerning the persistence of phonetic drift and L2 phonetic changes, findings at the L1 re-immersion time point suggest only a partial reversal of the L1 changes which occur over the RA, and all systemic L2 developments are sustained. Finally, over time, the distance between participants L1 and L2 vowels diminishes consistent with assimilation. Overall, these results start to paint a clearer picture of longitudinal phonetic drift by highlighting L2 phonetic development as a predictive factor of this L1 change, as well as underlining the existence of a relationship between the loss of L2 phonetic gains and the reversal of L1 phonetic drift upon returning home.

More broadly, these results have implications for L2 speech models and how sounds are represented in the minds of language learners. We argued that the production of L1 and L2 sounds is guided by a composite systemic representation that is characterized by the combined global acoustic distributions for both the native and foreign sound systems to which they are exposed over time. Future models of L2 speech learning may therefore not only need to be able to account for bidirectional cross-linguistic influence but also phonetic interactions both between individual segments and the wider sound systems to which those segments belong.

## Appendix A

### English Items

**Table:**

FLEECE	STRUT
I'll have **tea** cups	The rock **juts** out from the mountain
They **see** four criminals	Place the **cup** on the counter
Four **keys** on a ring	She **putts** down the green
The **feed** is outside	He **butts** heads with his brother
Their **feet** have to be measured	She **shuts** both doors at night
Two **bees** on the flower	He **guts** two fish per day

**Table:**

KIT	FOOT
He **fits** the bill	The **books** from the store
Remove the **pits** from the peaches	She **foots** the bill every time
She **hits** two homeruns at every game	Two **cooks** in the kitchen
There are first aid **kits** in all rooms of the building	He **took** four apples from the store
She **sits** on the sofa	Watch out for the fish **hooks** on the dock
He had a few **zits** on his chin	She **shook** the tree

**Table:**

DRESS	GOOSE
He **bets** two hundred	I'll have **two** cups
She **said** we could eat	Good things **do** come in small packages
A small **peck** on the cheek	We are building a chicken **coop** tomorrow
They built a **shed** in the backyard	The **food** is outside
The dog **jets** down the hallway	They **sue** four criminals
Two **debts** to resolve	I bought five **shoes** yesterday

**Table:**

TRAP	THOUGHT
He **bats** two hundred	He's **fought** a good fight
The **tack** in the wall fell out	She hasn't **poured** herself any water
Put the **cap** by the recycle bin	No one knows the **source** of magic
A small **pack** on the ground	My brother's **caught** a cold
The **back** of the store	They play **board** games at weekends
She is **sad** about the breakup	Close the **doors** behind you

**Table:**

LOT	BATH
The **pots** are in the kitchen	He doesn't run **fast** enough
There are **dots** on the floor	It's just a **part** of life
There are **cots** for the children	He doesn't **pass** the ball
A small **dock** in the harbor	My **card** is the highest
She **spots** four criminals	It's **dark** outside now
The court reporter **jots** down the case	I once ate a **shard** of glass

### French Items

**Table:**

/i/	/a/
Sa fille et son **fils** parlent lentement	Je veux **ta** pomme
Les tortues **vivent** longtemps	Mot de **passe** oublié
C'est une **cible** qui bouge	Mon **chat** joue avec mes amis
Elle **vise** un sanglier	Tu connais **sa** sœur
Vous **dites** qu'elle voulait nous voir	Je veux qu'il **sache** la vérité
On se fait la **bise** chaque matin	Je n'ai **pas** d'espèces

**Table:**

/y/	/œ/
Je n'ai pas **bu** de jus	Son chef d'**œuvre** est formidable
Je n'ai pas **pu** te montrer	Je voudrais un petit **œuf** bio
Il a **su** faire ses preuves	La coquille d'**œuf** fait partie de la recette
J'ai mangé **du** fromage	Une petite **œuvre** artistique
On peut le faire **vu** qu'il nous comprend	Elles **peuvent** danser ce soir
Si **tu** veux, il n'y a aucun soucis	Ils **peuvent** acheter quelque chose

**Table:**

/e/	/ɔ/
Il a **des** enfants	Je vais aller voir mon **pote** ce week-end
Tu as **tes** affaires	Un petit **os** du corps humain
Les contes de **fées** sont faciles à lire	Il aime son travail donc il **bosse** beaucoup
Il veut **ses** vêtements	Allez à la **poste** pour envoyer votre lettre
Tout ce que j'**ai** préparé	Son chapeau a une **poche** secrète
Laisse-le **chez** toi	C'était mon rêve de **gosse** de le voir

**Table:**

/ɛ/	/o/
Je voudrais **cette** pomme	Passons par la **côte** d'Azur
Les enfants **jettent** des pierres	Un **sot** qui fait rire
Le **chef** de cuisine	J'aime **Peau** d'Ane
Elle ne **cesse** de fumer	Mon **beau**-frère
Je veux qu'il **sèche** ses vêtements	Le chat **saute** sur moi
J'ai la **tête** ailleurs	Le **taux** de chômage

**Table:**

/ø/	/u/
Il a **deux** voitures	La soie est très **douce** et fine
C'est **peu** de fromage	Il faut que **tous** ces hommes mangent
Un musée comme **ceux** de Paris	Au **bout** de la salle
J'ai eu trois **feux** verts sur la route	J'aime les petites **pousses** de laitue
Je fais le **vœu** de ne plus fumer	C'est **vous** qui l'aimez
Les **jeux** d'enfants	C'est le **fou** qui s'est trompé

## Appendix B

**Table B1.** Testing and Participant Information.

**Location and date of first recording session** **(pre-RA)**	22: universities in the south of England (2–3 months pre-RA) 20: Lyon (within first 3 months of RA)
**Length of RA**	Mean months abroad: 6.3, range: 5.1–8.0
**Location and date of second recording session (post-RA)**	Within 2 months of returning to the L1 environment (online) Mean days: 17.6, range: 0–50
**Location and date of third recording session** **(L1 re-immersion)**	~10 months after returning to the L1 environment (online) Mean months of L1 re-immersion: 10.45, range: 5.6–12.2
**Location of RA**	40: France (Paris and Lyon for the vast majority) 1: Geneva, Switzerland 1: Dakar, in Senegal
**Type of RA**	25: University students 16: English teachers/assistants 1: Work placement
**French proficiency**	French Lextale vocabulary test (Brysbaert, 2013) *M*: 61.7%, range: 49.1%–88.4%
**Length of learning French**	Generally intermediate-advanced instructed learners *M*: 8.4 years, range: 0–15
**Previous naturalistic French input**	Days spent living in French-speaking country before the pre-RA data collection session: *M*: 8.5, range: 0–104
**L1 English variety**	Participants self-reported L1 variety 27: South (England) 8: The United States 4: Midlands (England) 2: Scotland 2: North (England)
**Other languages used on a regular basis?**	5: Spanish 2: Spanish and German 1: German 1: Russian

*Note.* RA: residence abroad.

## Appendix C

**Table:** Model 1 (Baseline F1 Comparison).

Fixed effects	Estimate	*SE*	*df*	*t*	Significance
**Intercept**	−1.08	0.03	125.37	−38.72	***
**Group** (NFS/ELoF Eng (pre-RA)	0.22	0.05	132.22	4.02	***
(NFS/ELoF Fren (pre-RA)	−0.05	0.01	55.54	−3.06	**

*Note.* NFS: native French speakers.

Formula: F1 Norm ~ Group + (1 | Speaker) + (1 | Word).

No. of observations: 5,510.

Random Intercepts: Word (117, Var = 0.08, *SD* = 0.28); Speaker (52, Var = 0.00, *SD* = 0.04).

Significance levels: ****p* < .001. ***p* < .01. **p* < .05. ^†^*p* < .1.

**Table:** Model 2 (Baseline F2 Comparison).

Fixed effects	Estimate	*SE*	*df*	*t*	Significance
**Intercept**	0.23	0.03	119.60	8.56	***
**Group** (NFS/ELoF Eng (pre-RA)	−0.07	0.05	123.85	−1.30	n.s.
(NFS / ELoF Fren (pre-RA)	−0.02	0.01	67.64	−1.46	n.s.

*Note.* NFS: native French speakers.

Formula: F2 Norm ~ Group + (1 | Speaker) + (1 | Word).

No. of observations: 5,510.

Random Intercepts: Word (117, Var = 0.08, *SD* = 0.28); Speaker (52, Var = 0.00, *SD* = 0.03).

Significance levels: ****p* < .001. ***p* < .01. **p* < .05. ^†^*p* < .1.

**Table:** Model 3 (F1 Change Over Time).

Fixed effects	Estimate	*SE*	*df*	*t*	Significance
**Intercept**	−0.86	0.03	122.35	−25.43	***
**Time Point** (Post-RA Eng/Pre-RA Eng)	−0.10	0.01	92.67	−9.73	***
(Post-RA Eng/L1 re-immersion Eng)	−0.03	0.01	29.38	−2.42	*
**Language** (English/French)	−0.24	0.05	120.95	−4.95	***
**Time Point × Language**					
(Post-RA/Pre-RA Fren) compared with (Post-RA/Pre-RA Eng)	−0.03	0.01	109.59	−2.56	*
(Post-RA/L1 re-immersion Fren) compared with (Post-RA/L1 re-immersion Eng)	0.01	0.01	31.52	.41	n.s.

*Note.* RA: residence abroad.

Formula: F1 Norm ~ Time Point * Language + (1 + Time Point * Language | Speaker) + (1 + Time Point | Word).

No. of observations: 13,178.

Random Intercepts: Word (117, Var = 0.07, *SD* = 0.26); Speaker (42, Var = 0.00, *SD* = 0.04).

Significance levels: ****p* < .001. ***p* < .01. **p* < .05. ^†^*p* < .1.

**Table:** Model 4 (F2 Change Over Time).

Fixed effects	Estimate	*SE*	*df*	*t*	Significance
**Intercept**	0.19	0.04	117.21	5.30	***
**Time Point** (Post-RA Eng/Pre-RA Eng)	0.00	0.01	67.95	−.30	n.s.
(Post-RA Eng/ L1 re-immersion Eng)	−0.01	0.01	52.25	−1.58	n.s.
**Language** (English/French)	0.05	0.05	117.43	0.89	n.s.
**Time Point × Language**					
(Post-RA/Pre-RA Fren) compared with (Post-RA/Pre-RA Eng)	0.01	0.01	80.47	0.84	n.s.
(Post-RA/L1 re-immersion Fren) compared with (Post-RA/L1 re-immersion Eng)	0.00	0.01	38.98	−0.13	n.s.

*Note.* RA: residence abroad.

Formula: F2 Norm ~ Time Point * Language + (1 + Time Point * Language | Speaker) + (1 + Time Point | Word).

No. of observations: 13,178.

Random Intercepts: Word (117, Var = 0.08, *SD* = 0.28); Speaker (42, Var = 0.00, *SD* = 0.02).

Significance levels: ****p* < .001. ***p* < .01. **p* < .05. ^†^*p* < .1.

**Table:** Model 5 (Change in Individuals’ Mean F1 Difference Between English and French Over Time).

Fixed effects	Estimate	*SE*	*df*	*t*	Significance
**Intercept**	0.24	0.01	40.58	28.99	***
**Time Point** (Post-RA Eng/Pre-RA Eng)	0.03	0.01	69.15	2.61	*
(Post-RA Eng/L1 re-immersion Eng)	−0.01	0.01	73.14	−0.57	n.s.

*Note.* RA: residence abroad.

Formula: F1 Distance (between English and French) ~ Time Point + (1 | Speaker).

No. of observations: 114.

Random Intercepts: Speaker (42, Var = 0.00, *SD* = 0.04).

Significance levels: ****p* < .001. ***p* < .01. **p* < .05. ^†^*p* < .1.

## Notes

1. Note, however, that any differences between the L1 Quichua-L2 Spanish speakers and the L1 Spanish speakers are not particularly due to recent L2 experience and, therefore, may only be loosely construed as phonetic drift according to its definition in the present study.

2. It is unlikely that this group of speakers evidenced crucial differences in their French productions from functionally monolingual French speakers given that little vocalic drift is reported within this time frame without explicit training (e.g., Lang & Davidson, 2019). As such, we believe these speakers form a suitable reference group for the present purposes.

3. The item “putt” was removed from the STRUT tokens due to many ELoF mistakenly uttering “put” across all three time points.

4. Due to COVID-19, the second and third time point were conducted virtually, and careful checks of the recordings were carried out for any recording failings or substantial background noise. No vowel tokens had to be excluded as a result. To perform further checks, a separate analysis of only the most reliable formant measures, as determined by bandwidths below 200 Hz, was conducted. Results for this subsample were consistent with the wider sample, suggesting the phonetic changes observed in this research are not motivated by differences in recording quality.

5. 164.5 Hz was the midpoint between population F0 means for men, who are known to have longer vocal tracts, and women, who have on average shorter vocal tracts (Pisanski et al., 2014).

6. For example, in Models 3 and 4, Language was included as a random slope for Speaker but not for Word because the same speakers provided data for both languages, but Language was manipulated between, not within, Word (Linck & Cunnings, 2015, p. 196).

7. The one participant undertaking a work placement was excluded from this analysis.

8. Although this relationship was only observed in Kartushina and Martin (2019), a potential outlier was not excluded from analyses.
